# Medical Science Liaisons (MSL) in Africa: a perspective

**DOI:** 10.11604/pamj.2019.33.313.15143

**Published:** 2019-08-20

**Authors:** Dwayne Koot, Alicia McMaster, Monique Nel, Alet van Tonder, Alan Barret, Poobalan Naidoo

**Affiliations:** 1 Sanofi Medical Affairs, Midrand, South Africa

**Keywords:** Africa, Medical Science Liaison (MSL), relationship, responsibilities, scientific exchange, challenges, opportunities

## Abstract

The role of a Medical Science Liaison (MSL) is of growing importance to pharmaceutical, biotechnology, diagnostic and medical device companies. Through scientific engagement MSLs add value to clinical practice, ultimately benefiting patients. The MSL role is dynamic and encompasses in-depth product and disease knowledge together with the ability to communicate relevant, unbiased scientific information concisely and timely. Tasks are focused on contributing towards the advancement of medical knowledge, scientific data generation and dissemination. Professional relationships are developed, fostering collaboration between external experts and typically the medical affairs departments of pharmaceutical companies through a credible liaison. Through such relationships, critical insights are shared that shape the development pipeline, promote successful clinical translation and guide the market deployment strategy of therapeutic interventions through-out their life cycle. Despite the rising number of MSLs in the field and the implicit medical value of the role, there remains a lack of understanding for what the roles of a MSL entails. In Africa, where exponential growth of the pharmaceutical industry is expected, the number of MSLs will increase rapidly. Given the complexities of the African continent, the MSLs in this burgeoning environment will face various challenges including remote locations, time-constraints, regulatory and bureaucratic hurdles and importantly physician misperception of the MSL role that collectively may thwart the goal of meaningful scientific engagement; but these challenges can be surmounted through astute proactive planning and utilization of opportunities including digital communication strategies.

## Perspective

Africa comprises over 20% of the world's land mass and has a population in excess of a billion. Africa bears the greatest burden of disease in the world and the lowest life expectancy [1], however the pharmaceutical industry derives only ~2% of their global market share from Africa [2]. The reasons for the aforementioned discrepancies are multifactorial, including evolving regulations, infiltration of counterfeit medicines, reliance upon or preference for traditional medicines, supply chain constraints and socio-economic conditions. Owing to the large population, the prevalence of infectious disease (including HIV/AIDS, malaria and tuberculosis) and growing non-communicable as well as lifestyle disease, the need for innovative therapies to lessen morbidity and mortality is substantial. The American and European pharmaceutical market growths are tending to plateau; conversely growth in the developing world is fast and the African pharmaceutical industry is expected to be worth $40-60 billion by 2020 [3]. This growth in volume, along with new launches of sophisticated therapies (including biologics, rational combinations and intelligent formulations) requires innovative pharmaceutical companies to increasingly use skilled Medical Science Liaisons (MSLs) to generate and disseminate pertinent scientific data. MSLs also known as Regional Medical Advisors amongst a host of other alternate titles [4, 5] are generally highly credentialed (MD, PhD, pharmacists, and in some instances medical specialists) [5] and can have peer to peer discussions with external experts. Although the MSL role is considered relatively new in Africa, the role begun in developed countries in the late 1960's and although still evolving is currently advanced [6, 7]. Recently, for the first time the roles and expectations for a MSL have been somewhat harmonized through the publication of an international guideline document by the Medical Science Liaison Society (MSLS) [5]. The purpose of this paper is to describe the role and responsibilities of MSLs (as collated from various publications, societies, round table discussions and leaning on the experiences of the authors); to describe the MSL-physician relationship (the nature, content and value of this engagement); to describe the differences between the MSL and other field based functions and to outline future needs and directions to maximally utilise the MSL resource.

**The role and responsibilities of a MSL:** core competencies of a MSL include: expert knowledge of therapeutic area, products and health care systems; internal and external stakeholder management (engagement strategy); and data/information generation or compilation and dissemination. A MSL is a subject matter expert and an educational resource to the healthcare community of current evidence based medical science pertaining to a particular therapeutic area or drug class. For many, the primary activity of the MSL is to foster and develop professional relationships with external experts (key opinion leaders) and to facilitate the exchange of scientific information, perceptions, needs and opportunities [4]. Further tasks include training (internal and external stakeholders), pharmacovigilance, pharmacoeconomic modelling, medical writing, scientific presentations, and regulatory support, gathering competitive intelligence, medico-marketing support and partnering with health ministries to address needs. The scope for a MSL to contribute towards advancing medical science is substantial and there is opportunity to facilitate, encourage, implement and support research projects through collaboration between principal investigators and the pharmaceutical industry. The MSL’s role is dynamic and in order to be effective must be strategically aligned with the objectives of the organisation particularly during pre-, peri- and post-launch when the value of an ethical relationship with influential, peer respected external experts may be a critical success factor [4-8]. The defining responsibility and critical competency contributing towards success is communication [9, 10], both externally and internally, and in so doing fulfilling the role of liaison between the pharmaceutical company and medical community at large. The ability to professionally exchange and interpersonally connect on scientific matters in an ethical, unbiased, articulate, concise and compelling manner is necessary to advance medical knowledge and add value to patient care. Table 1 lists the key MSL competencies, roles and responsibilities.

**Table 1:** Medical Science Liaisons competencies, role and responsibilities

Therapeutic area, product and regulatory knowledge
Data generation and dissemination
Health care system knowledge
Stakeholder strategy and planning Competitive intelligence
Internal and external stakeholder engagement
Scientific relationship building and collaboration with external experts

**The MSL-physician relationship:** the MSL role is distinct and separated from commercial (sales and marketing) functions by company standard operating procedures and policies. The goal of the interaction is not promotional and this should be clear from the tone and content of all interactions. Therefore all interactions with thought leaders should be patient centric, ethical, fair and balanced [7], and in compliance with local and international regulations. The goal of the MSL is to be an unbiased subject matter expert- a peer with whom a key external expert can engage to obtain detailed information regarding the ever increasing armamentarium and complexity of therapies (particularly with the increase in the use of biologicals and a growing trend towards personalised medicine). A MSL serves to help interpret and provide clarification to aid understanding of new clinical safety, efficacy and health economic data. This relationship aims to add value to clinical management through being part of the extended multidisciplinary team. The scientific exchange, as the basis of the relationship, is two-way and information is collected from the field that can inform and direct future activities of the pharmaceutical company to better address unmet local needs [11].

**MSL differentiators from other field based personnel:** the goal of a MSL is to advance the standard of care and improve patient outcomes [4]. Sales are not a metric of MSL performance, thus there is no pressure to sell a product, therefore limiting the probability of dissemination of biased information. Furthermore, the credentials (MSLs may have advanced medical related degrees and are often registered with medical councils) and extensive scientific training is another factor that often differentiates MSLs from professional sales representatives (PSRs). They do not detail as per traditional PSRs but rather engage strategically at peer to peer level. MSLs are able to analyse both the strengths and the limitations of studies thus ensuring that drug utilization is based on all available safety and efficacy data. This builds trust and grows into an advisory capacity that in turn also gives the MSL greater access to external experts (EE). Where teaching and infrastructure is limited, the MSL will serve as an important source of information but also as an emotionally invested and understanding peer that the EE can use as a "sound board" to voice interests and share concerns. The MSL builds scientific relationships nationally and internationally and thus can serve to establish networks and grow the connection between EEs. MSLs attend international congresses and thus have first-hand exposure to latest scientific advancements that can be then disseminated in local environments. PSR's seldom attend international congresses but do of course rely on the internal educational resource provided by the MSL. The MSL role includes data generation and dissemination through clinical trials, manuscripts, congress presentations and scientific meetings. Furthermore, external stakeholder engagements are not limited to clinicians but medical organisations and societies, funders and patient advocacy groups. Table 2 lists the key differentiators between the MSL and other field based personnel.

**Table 2:** Medical Science Liaisons differentiators

Sales is not a metric
Highly credentialed and extensive scientific training
National and International networks
Peer to peer interaction on specific subject matter and do not detail on products
Exposure to state of the art medical developments at international congresses Data generation and dissemination through clinical trials, manuscripts, congress presentations and scientific meetings Ability to engage with medical societies, funders, patients advocacy groups, etc.

**Challenges faced in Africa:** in our opinion, challenges faced by MSLs in Africa are likely to be similar to other developing regions e.g. India, Pakistan and Brazil. Unfortunately, there is a paucity of information on challenges faced by MSLs in developing regions and we thus cannot support our view with evidence-based literature. The MSL concept remains poorly understood by external experts in Africa (based on anecdotal data from senior medical advisors who have managed MSLs throughout Africa and current MSLs operating in these settings). Limited awareness of the role of a MSL, both internally and externally, remains a challenge. In European and American markets where the MSL role is well established, medical practitioners request appointments with MSLs to discuss scientific concepts. However, in Africa many medical practitioners are not familiar with the value of this role and consequently MSLs compete with PSRs for the limited time slots allocated at practices for interactions with the pharmaceutical industry. Furthermore, while PSRs require brief appointments with medical practitioners to deliver their message whereas a MSL requires an extended appointment in order to discuss complex scientific concepts fully. As few practices are currently able to accommodate lengthy appointments, MSLs are often faced with the task of condensing valuable conversations or seeking alternative platforms to engage with external experts. The MSL is sometimes the only company employee working in certain African countries and they are often expected to assist with general company operational tasks which do not necessarily fall within the remit of their function. This dilutes the effectiveness of the MSL in these settings and may adversely affect the confidence of the MSL. As the role of a MSL requires communication, there is a human element involved that requires good social, emotional and cultural intelligence. Africa consists of many countries with vastly different cultural, political and regulatory systems. One approach to effective scientific exchange may not be suited to all regions. There may be substantial differences in the standard of care and medical practice between different regions, even within the same country. Considering the sparsity of infrastructure and the geographical distances between centres of clinical excellence, the field based role of a MSL will invariably require extensive national and international travel. Whilst a national MSL would be of value, retention of high calibre individuals remains challenging. The MSL will consequently have to be comfortable with travel and be sensitive to the norms, customs and language of various regions so as to quickly grow rapport, build trust and be able to engage at the scientific level as peers working towards the same goal of advancing healthcare for patients. Given the large geographical spread and the relatively poor infrastructure, the MSL experiences challenges in the frequency of face-to-face visits. Identification of peer respected opinion leaders with whom meaningful relationships can be developed is vital. Although the internet may be used to engage doctors in difficult to reach places, internet speeds and available supporting infrastructure may be limited. At present digital technology platforms have not effectively delivered key scientific messages. Africa has the lowest physician-to-population ratio of only 2.7 per 10 000 population in contrast to global average of 13.9 and 32.1 for Europe. Pharmaceutical personnel density is 0.8 per 10 000 for Africa in contrast to 4.5 and 6.8 for the global average and Europe respectively [1]. The healthcare sector is understaffed making time efficient communication and well rationalised strategic plans all the more important.

**The future:** the landscape of healthcare in Africa is changing. With the advent and growth of digital technology, innovative connectivity tools provide the opportunity to communicate rapidly, near constantly and successfully maintain and grow relationships. Although there are connectivity issues currently, these are likely to be addressed in the near future, allowing rollout of effective digital technology platforms. Such technological innovations-specifically connectivity tools, may have a significant impact upon developing and maintaining relationships with external experts, patient advocacy groups, health ministries and non-governmental organisations with the intent of educating or conducting research. There is currently a paucity of data with respect to MSLs and perceptions by external and internal stakeholders within Africa. Future research is required to get quantitative and qualitative insight that will assist with developing a road map to ensure effective use of the MSL function in Africa. Going forward, it is hoped that the pharmaceutical industry within Africa will harmonise the MSL competencies, roles and responsibilities across the industry to limit misunderstanding and grow the status of the MSL.

## Conclusion

As innovative, complex and precision therapies emerge from development pipelines, and as the pharmaceutical industry grow in Africa, MSLs are suited to fulfil unmet data needs in an ethical and compliant way. Ultimately, the success of the MSL role in achieving pre-defined, non-commercial focused performance metrics will be determined by the extent to which individual organisations identify and learn from specific regional challenges and are capable of innovating or embracing approaches to maximize meaningful scientific engagement.

## Competing interests

The authors declare no competing interests.
